# Fluorescent carbon dots with excellent moisture retention capability for moisturizing lipstick

**DOI:** 10.1186/s12951-021-01029-6

**Published:** 2021-09-30

**Authors:** Chen Dong, Mingsheng Xu, Shuna Wang, Menghui Ma, Ozioma U. Akakuru, Haizhen Ding, Aiguo Wu, Zhengbao Zha, Xuemei Wang, Hong Bi

**Affiliations:** 1 School of Chemistry and Chemical Engineering, Key Laboratory of Environment Friendly Polymer Materials of Anhui Province, Anhui Key Laboratory of Modern Biomanufacturing, Anhui University, Hefei, 230601 China; 2 Cixi Institute of Biomedical Engineering, International Cooperation Base of Biomedical Materials Technology and Application, CAS Key Laboratory of Magnetic Materials and Devices, Zhejiang Engineering Research Center for Biomedical Materials, Ningbo Institute of Materials Technology and Engineering, Chinese Academy of Sciences, Ningbo, 315201 China; 3 Advanced Energy Science and Technology Guangdong Laboratory, Huizhou, 516003 China; 4 School of Food and Biological Engineering, Hefei University of Technology, Hefei, 230009 China

**Keywords:** Carbon dots, Nano-additives, Moisture retention, Moisturizing appreciation rate, Skin care

## Abstract

Long-lasting moisture retention is a huge challenge to humectants, and effective methods or additives for promote these functions are limited, especially nano-additives. Carbon dots (CDs) have attracted increasing research interest due to its ultra-small size, excellent optical properties and low toxicity, etc. However, most of researches have been focused on the photoexcited CDs and its subsequent photophysical and chemical processes, such as photoluminescence, photodynamic, photothermal and photocatalytic behavior. The intrinsic chemo-physical properties of the pristine CDs are not fully explored. Here, we report an excellent moisture retention capability of a new carmine cochineal-derived CDs (Car-CDs) for the first time. The relationship between the structure of Car-CDs and its moisture retention capability is revealed. More interestingly, the effective applications of Car-CDs in moisturizing lipstick are demonstrated. This work expands the research and application of CDs into a broad, new area, potentially in skin care.

**Supplementary Information:**

The online version contains supplementary material available at 10.1186/s12951-021-01029-6.

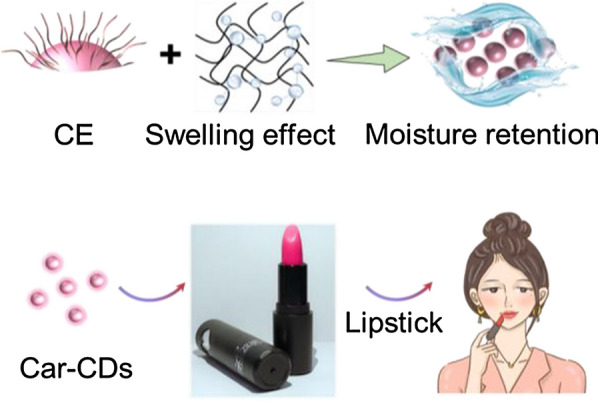

## Introduction

Humectants have been widely used in food, medicine, and cosmetics owing to their excellent water stabilization capacity during storage and transportation of products [1]. Commonly, hydrophilic polyols such as glycerin and propylene glycol are used for moisturizing additives to improve the biocompatibility of the matrix and endow cosmetic products with moisture retention properties. However, long-lasting moisturizing functions is one of the issues for traditional humectants, which makes it difficult to meet industrial demand. Recently, several studies have devoted to the extraction of natural polysaccharides and exploring the potential applications in skin care [2]. Although natural products are renewable, the problems of complicated material preparation and low yield severely limit their practical applications.

Carbon dots (CDs), with excellent tunable optical properties and good biocompatibility, show great promise as sensors, photocatalysis, photoelectric devices, and multifunctional theranostic systems, among others [3–10]. However, extensive studies on CDs have been focused on improving synthetic strategy, surface engineering, and photoluminescence mechanism [11–16]. Thus, deeply explore the intrinsic chemo-physical properties of CDs is essential for innovative application in the future. It is well-known that freshly freeze-dried CDs are highly hygroscopic. This ability is closely associated with its surface groups, which consists largely of hydroxyl and carboxyl. Taking into account the excellent water solubility and abundant surface functional groups of CDs, it can be used as a potential humectant. Nevertheless, the research on CDs-based moisturizing systems remains unexplored.

Here, a new type of carmine cochineal-derived CDs (Car-CDs) is synthesized via one-pot solvothermal method. Among commonly-used synthesis methods, the solvothermal synthesis has many advantages such as being economical, easy to handle and with a high efficiency to synthesize CDs from diverse carbon-based precursors. Importantly, we have systematically studied the moisturizing activity of CDs for the first time. In addition, the moisture retention function of Car-CDs in human skin was further evaluated with moisture measurement value method. More interestingly, we use the Car-CDs as a nano-additive in the preparation of moisturizing lipstick, which suggests its potential beneficial application in health or skin care and cosmetics.

## Results and discussion

**Fig. 1:**
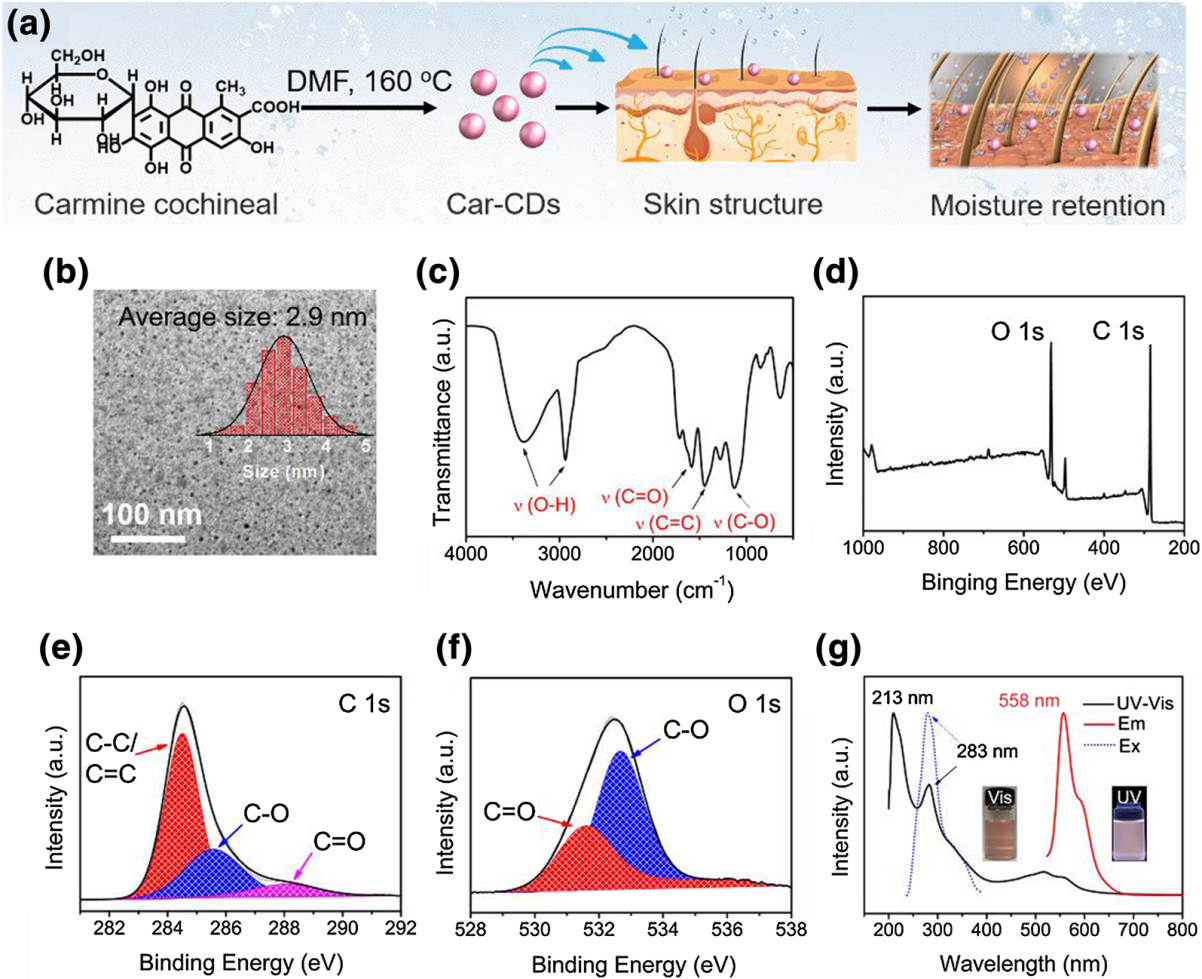
(a) Schematic illustration of the preparation procedure and moisture retention capability for Car-CDs. **b** TEM image, **c** FT-IR, and **d** XPS spectra of Car-CDs. **e** HR XPS C1s and **f** O1s spectra of Car-CDs and fitting results. **g** UV–vis absorption and PL spectra of Car-CDs

A detailed description of the methods and experiments is included in the Additional file 1. As shown in Fig. 1a, through a solvothermal method from carmine cochineal in *N, N*-dimethylformamide (DMF) at 160 °C for 6 h [17], the bright pink-emissive CDs (Car-CDs) are synthesized. Figure 1b shows that the transmission electron microscopy (TEM) image of the as-prepared Car-CDs are uniform in shape with an average diameter of 2.9 nm. An average lattice spacing of 0.21 nm, which is corresponding to the (100) facet of graphite [18], can be clearly observed in the high-resolution TEM image of Car-CDs (Additional file 1: Figure S1). In addition, the Raman spectrum of Car-CDs displays two signals at 1359 and 1585 cm^**−**1^ for D and G bands, respectively (Additional file 1: Figure S2). Commonly, the *I*_D_/*I*_G_ ratios are widely used to evaluate the quality of carbon materials [19]. The ratio of *I*_D_/*I*_G_ is about 0.63, indicating a high graphitization degree in Car-CDs [20].

The chemical structure and surface functional groups of the Car-CDs were determined by Fourier transform infrared (FT-IR) spectroscopy and X-ray photoelectron spectroscopy (XPS). As shown in FT-IR spectrum (Fig. 1c), the two absorption bands at 3285 and 2937 cm^**−**1^ can be attributed to the stretching vibrations of −OH [21]. Three strong absorption peaks located at 1589, 1436, and 1123 cm^**−**1^ correspond to the stretching vibrations of C = O, C = C and C–O bonds, respectively [22, 23]. The XPS further confirm the above-mentioned FT-IR results. As shown in Fig. 1d, the Car-CDs mainly consist of C (285.1 eV, at.% = 74.9) and O (531.4 eV, at.% = 25.1) elements. In Fig. 1e, the high-resolution C 1 s spectrum reveals three peaks at 284.5, 285.6, and 288.2 eV assigned to C**–**C/C = C, C**–**O, and C = O, respectively [24, 25]. The HR XPS O 1 s spectrum has two peaks, which are attributed to C = O (531.6 eV) and C**–**O (532.7 eV) bonds, respectively (Fig. 1f) [26].

Subsequently, optical properties of Car-CDs were investigated. Figure 1g shows the absorption and emission spectra of Car-CDs in methanol. The absorption peaks at 213 and 283 nm are ascribed to *π–π*^⁎^ and *n–π*^⁎^, respectively [27]. The corresponding photoluminescence (PL) spectrum shows a emission (558 nm) under UV light excitation (283 nm), and their absolute quantum yields (QY) are measured to be 7.87% (Additional file 1: Figure S3). Contrarily, carmine cochineal exhibit almost no emission in solution under UV irradiation (Additional file 1: Figure S4). Besides, Car-CDs presented significant excitation-independent under different excitation wavelengths [28, 29] (Additional file 1: Figure S5), and the PL lifetime at 558 nm is calculated to be 3.86 ns (Additional file 1: Figure S6).

**Fig. 2:**
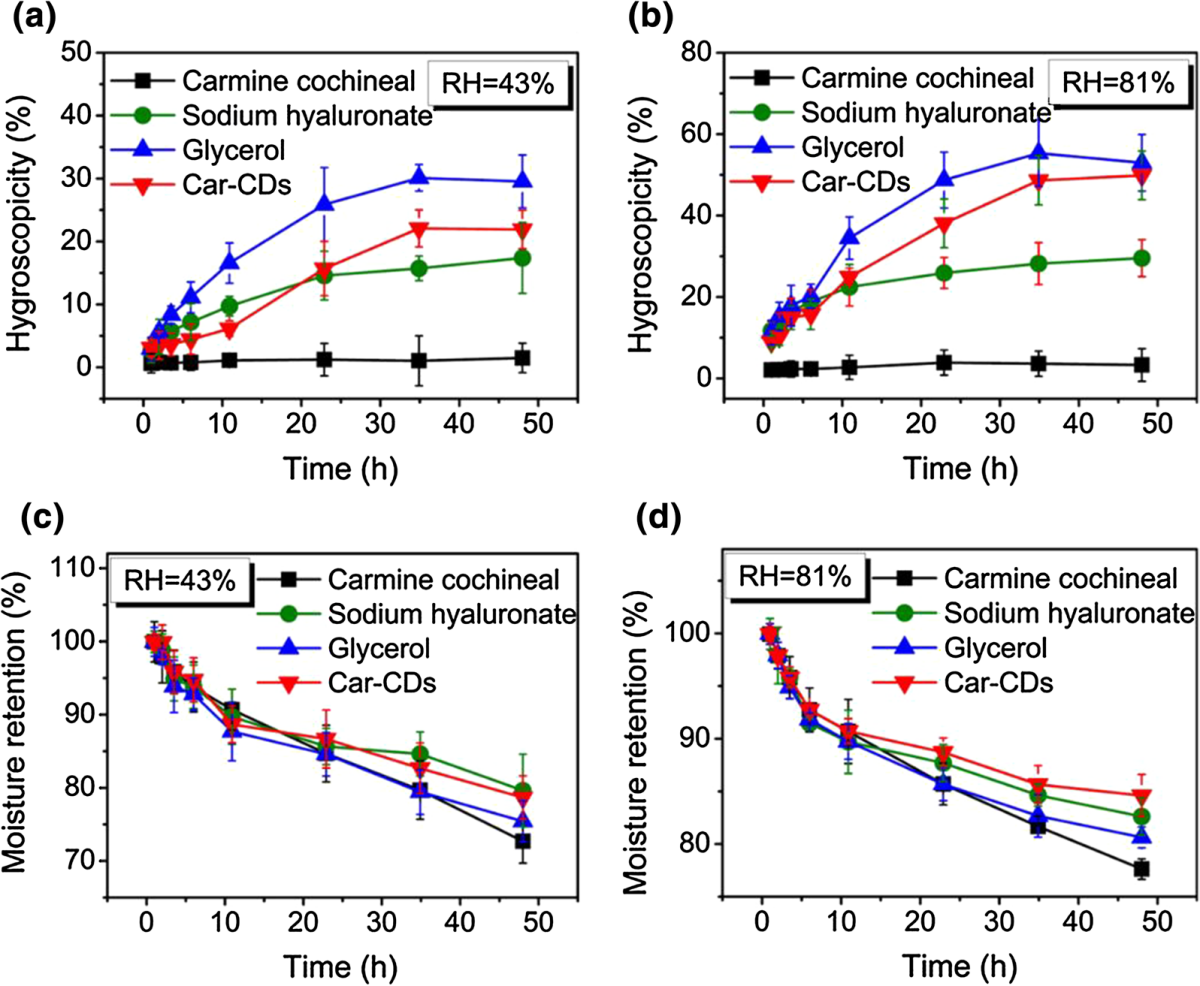
The relationship between hygroscopicity and time at RH = 43% **a** and RH = 81% **b** for different samples. The relationship between moisture retention and time at RH = 43% **c** and RH = 81% **d** for different samples

The moisture retention activities of Car-CDs were further assessed according to the established method [30, 31]. As shown in Fig. 2a, b, Car-CDs exhibit a dose-dependent trend in moisture-absorption ability under different humidity conditions [32]. Moreover, the moisture-absorption ability of Car-CDs at high humidity condition is equivalent to that of glycerin, frequently used as a hygroscopic agent. Similarly, the moisture retention rate of Car-CDs within 48 h at relative humidity of 43% and 81% are 78% and 84%, respectively (Fig. 2c, d).

**Fig. 3:**
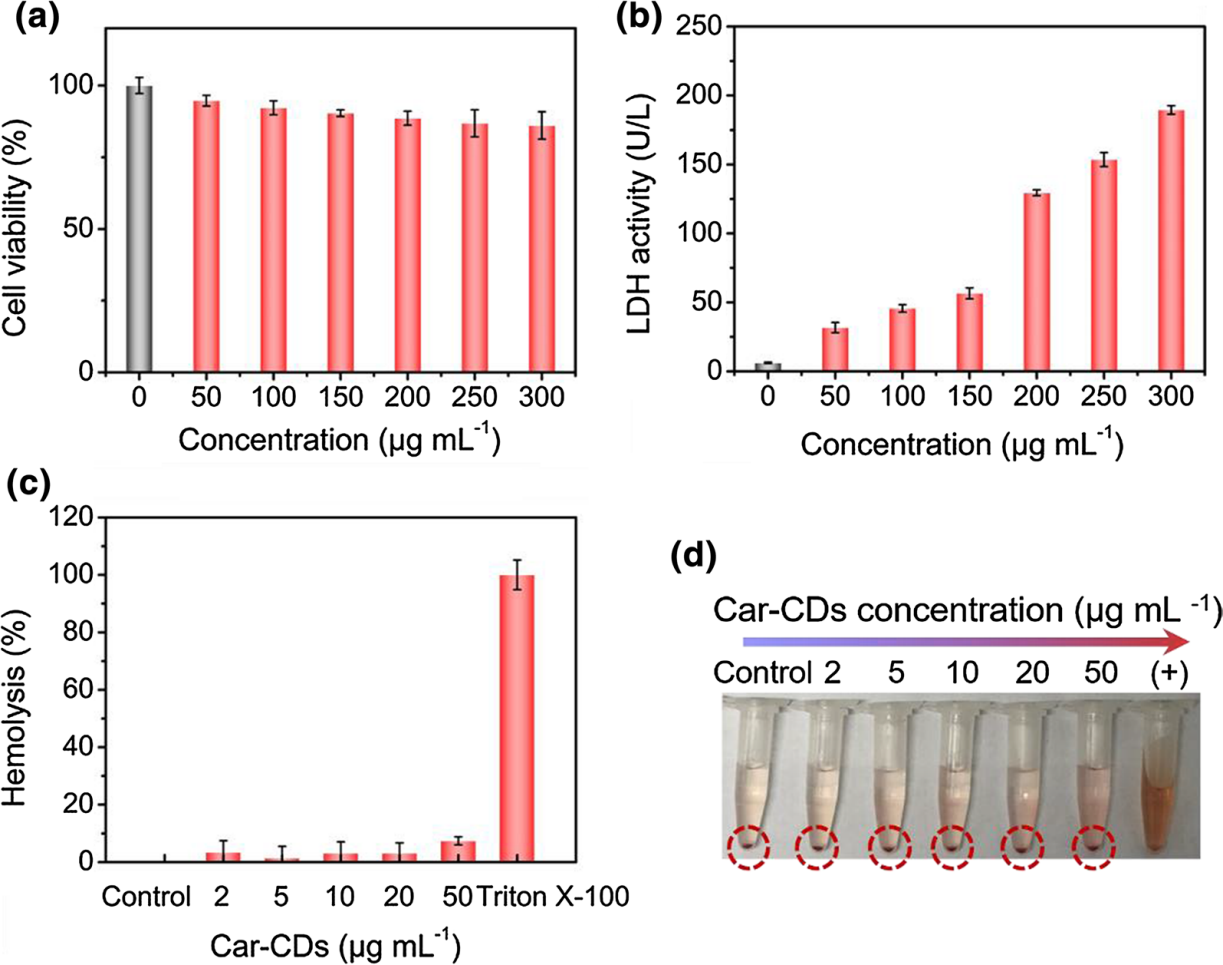
**a** MTT results of HUVEC cells co-incubated with various concentrations of Car-CDs for 24 h. **b** LDH release from the HUVEC cells treated with Car-CDs at various concentrations. **c** Hematological evaluation and **d** photographs of RBCs treated with different dosages of Car-CDs

To assess the potential applications of as-prepared Car-CDs in biological systems, cytotoxicity and hemocompatibility tests were performed to investigate their biocompatibility in vitro [33]. The MTT results show that more than 80% HUVEC cells still remain even with the concentration of Car-CDs reaching 300 μg mL^**−**1^, which agrees well with previous investigations [34] (Fig. 3a). As shown in Fig. 3b, the release of LDH in HUVEC cells exhibit a concentration-dependent effect [35], and the maximum release was less than 300 U L^**−**1^. Besides, the compatibility of Car-CDs with blood was evaluated by hemolysis assay. It can be seen that Car-CDs causes no significant hemolysis, and the maximum hemolysis rate is 5.2% (Fig. 3c, d). These results indicate that the hemolytic toxicity of Car-CDs to red blood cells (RBCs) is relatively low, which could allow for further blood applications [36].

**Fig. 4:**
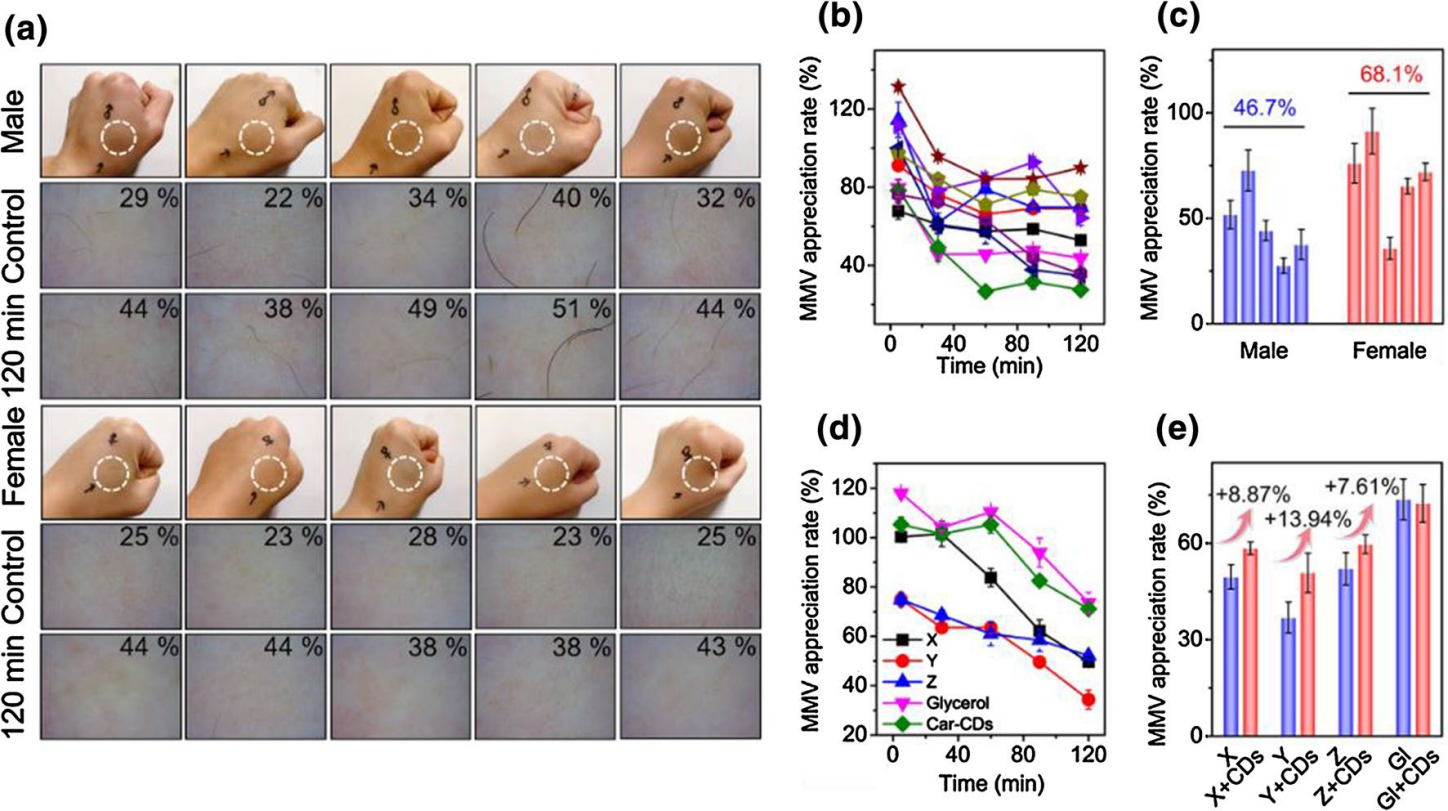
**a** Changes of moisture content in the hand skin of different volunteers after applying Car-CDs. **b** The change curve of hand skin moisture content after different volunteers applied Car-CDs. **c** Comparison of skin MAR between males and females after applying Car-CDs. **d** The effect on skin MAR of different samples. **e** The effect on skin MAR of different moisturizing products incubated with Car-CDs

Inspired by the appreciable moisture retention effect in vitro, the moisturizing performance of Car-CDs on human skin was further evaluated [37]. Ten volunteers were recruited to apply Car-CDs solution to their hands, and the changes in moisture content before and 2 h after the application was compared. It can be observed in Fig. 4a, b that the skin moisture of hands of different volunteers after applying the Car-CDs for 2 h improves to different degrees. Although the moisture content decreased continuously in the volunteers’ hand area, the overall trend is relatively stable, and the improvement rate of moisture is stable in the range of 27–89%. Interestingly, the results show that females have a significantly higher moisturizing appreciation rate (MAR) than males (Fig. 4c). Subsequently, the moisturizing appreciation rates of three other moisturizing products on the market (named X, Y, Z) and glycerol were evaluated. As shown in Fig. 4d, the efficiency of Car-CDs is comparable to that of glycerol and significantly higher than those of several commercially available moisturizing products. Unexpectedly, Car-CDs can also increase the MAR of several other moisturizing products accordingly. As shown in Fig. 4e, the addition of Car-CDs can significantly increase the MAR of X, Y and Z with the maximum increase of 13.94%. Therefore, these results demonstrate Car-CDs as nano-additives of high efficiency that possess moisture retention effects.

**Fig. 5:**
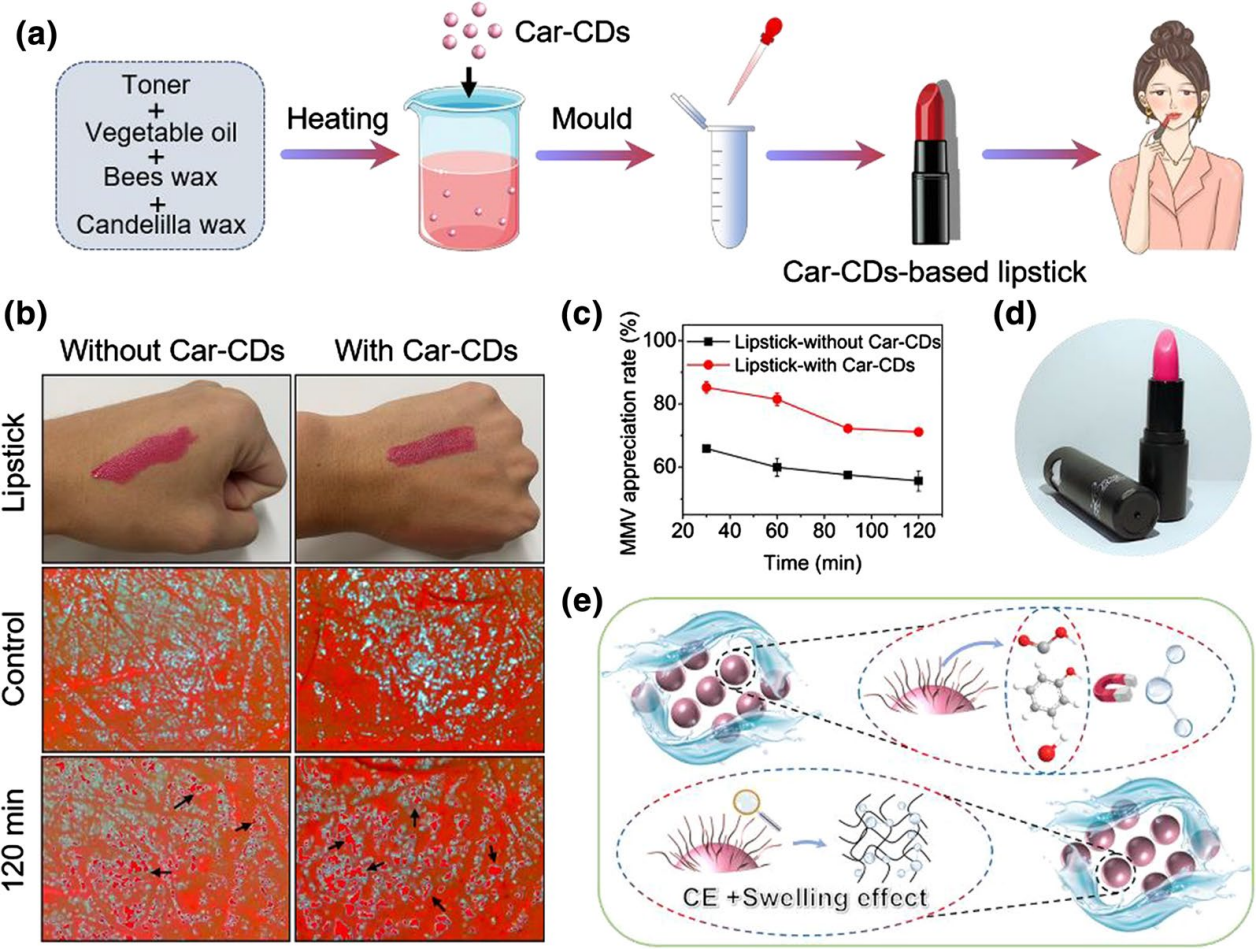
**a** Schematic illustration of the preparation procedure for Car-CDs-based moisturizing lipstick. **b** Changes of moisture content in the hand skin after applying moisturizing lipstick. **c** The change curve of hand skin moisture content applied moisturizing lipstick. **d** Photograph of the prepared moisturizing lipstick. **e** Illustration of the possible mechanism of moisture retention capability of Car-CDs

Excitingly, we successfully applied Car-CDs to moisturizing lipsticks as shown in Fig. 5a, d. Subsequently, the moisturizing effect of Car-CDs based lipstick on human skin were further evaluated. As shown in Fig. 5b, after volunteer applying Car-CDs-based moisturizing lipstick, the moisture retention on the skin of their hands was significantly improved. It can be intuitively observed in Fig. 5c that the MAR of Car-CDs-based lipstick is more excellent. Taking all of the experimental data together, we proposed a possible mechanism for moisture retention capability of Car-CDs. It is well known that hydrophilic groups on the surface of CDs can easily form hydrogen bonds with water molecules, thereby exhibiting moisture absorption properties. For this reason, we evaluated whether the CDs prepared by citric acid (CA-CDs) have a moisturizing effect. As shown in Additional file 1: Figure S7, CA-CDs has no significant moisture retention ability under different humidity conditions, indicating that hydrogen bonding should not be the main reason for the moisture retention performance. According to relevant literature reports [38], CDs possess a crosslink-enhanced (CE) effect, and the ability of their surface functional groups to bind water molecules during the cross-linking process may be further improved. In addition, the surface functional groups of CDs have polymer-like properties [39–41], and can effectively lock water molecules through water absorption and swelling (Fig. 5e) [42]. Since the unique moisturizing properties caused by the combination of CE and polymer swelling effect, CDs has great potential in skin care and cosmetics.

## Conclusions

In summary, we have developed a new type of Car-CDs with excellent moisture retention capability. Subsequent human skin tests confirmed that the Car-CDs were as effective as commercial moisturizers. Additionally, Car-CDs can also be used as nano-additives to enhance the moisturizing ability of other moisturizers. Our proposed mechanism is that the special CE effect is combined with the macromolecule swelling effect on the surface of Car-CDs, which are closely bound with the surrounding water. Thereafter, the Car-CDs were successfully used as a nano-additive in the preparation of moisturizing lipstick, which demonstrates its potential application in health or skin care and cosmetics.

## Supplementary Information

**Additional file 1: Fig. S1.** High-resolution TEM image of Car-CDs. **Fig. S2.** Raman spectra of Car-CDs (λ_ex_ = 532 nm). **Fig. S3.** Absolute fluorescence quantum yield of Car-CDs in methanol. **Fig. S4.** PL emission spectra of the Car-CDs and carmine cochineal under different conditions. **Fig. S5.** PL emission spectra with different excitation wavelengths of Car-CDs in methanol. **Fig. S6.** PL lifetime of Car-CDs. **Fig. S7.** The relationship between hygroscopicity and time at RH = 43% **a** and RH = 81% **b** for different samples. The relationship between moisture retention and time at RH = 43% **c** and RH = 81% **d** for different samples.

## Data Availability

All data generated or analyzed during this study are included in this article.

## Abbreviations

CDs: Carbon dot
Car-CDs: Carmine cochineal-derived CDs
DMF: *N*, *N*-Dimethylformamide
TEM: Transmission electron microscopy
FT-IR: Fourier transform infrared
XPS: X-ray photoelectron spectroscopy
PL: Photoluminescence
QY: Quantum yields
LDH: Lactate dehydrogenase
MTT: 3-(4,5-Dimethylthiazol-2-yl)-2,5-diphenyltetrazolium bromide
RBCs: Red blood cells
MAR: Moisturizing appreciation rate
CE: Crosslink-enhanced

## References

[1] Lin C, Cui H, Wang X, Wang H, Xia S, Hayat K, Hussain S, Tahir MU, Zhang X (2020). Regulating water binding capacity and improving porous carbohydrate matrix's humectant and moisture proof functions by mixture of sucrose ester and polygonatum sibiricum polysaccharide. Int J Biol Macromol.

[2] Song L, Xie W, Zhao Y, Lv X, Yang H, Zeng Q, Zheng Z, Yang X (2019). Synthesis, antimicrobial, moisture absorption and retention activities of kojic acid-grafted konjac glucomannan oligosaccharides. Polymers.

[3] Sharma A, Das J (2019). Small molecules derived carbon dots: synthesis and applications in sensing, catalysis, imaging, and biomedicine. J Nanobiotechnol.

[4] Dordevic L, Arcudi F, Prato M (2019). Preparation, functionalization and characterization of engineered carbon nanodots. Nat Protoc.

[5] Li C, Du X, Shi Y, Huang J, Wang Z, Zhang X (2019). Carbon nanodots enhance and optimize the photoluminescence of micro-spherical YBO_3_:Eu^3+^ phosphors. J Alloys Compd.

[6] Geng X, Sun Y, Guo Y, Zhao Y, Zhang K, Xiao L, Qu L, Li Z (2020). Fluorescent carbon dots for in situ monitoring of lysosomal ATP levels. Anal Chem.

[7] Shen CL, Lou Q, Liu KK, Dong L, Shan CX (2020). Chemiluminescent carbon dots: Synthesis, properties, and applications. Nano Today.

[8] Xu A, Wang G, Li Y, Dong H, Yang S, He P, Ding G (2020). Carbon-based quantum dots with solid-state photoluminescent: mechanism, implementation, and application. Small.

[9] Ai L, Yang Y, Wang B, Chang J, Tang Z, Yang B, Lu S (2021). Insights into photoluminescence mechanisms of carbon dots: advances and perspectives. Sci Bull.

[10] Li B, Zhao S, Huang L, Wang Q, Xiao J, Lan M (2021). Recent advances and prospects of carbon dots in phototherapy. Chem Eng J.

[11] Wang Z, Yuan F, Li X, Li Y, Zhong H, Fan L, Yang S (2017). 53% Efficient red emissive carbon quantum dots for high color rendering and stable warm white-light-emitting diodes. Adv Mater.

[12] Wang L, Li W, Yin L, Liu Y, Guo H, Lai J, Han Y, Li G, Li M, Zhang J, Vajtal R, Ajayan P, Wu M (2020). Full-color fluorescent carbon quantum dots. Sci Adv.

[13] Zhang X, Chen C, Peng D, Zhou Y, Zhuang J, Zhang X, Lei B, Liu Y, Hu C (2020). pH-responsive carbon dots with red emission for real-time and visual detection of amines. J Mater Chem C.

[14] Wang Z, Dong X, Zhou S, Xie Z, Zalevsky Z (2021). Ultra-narrow-bandwidth graphene quantum dots for superresolved spectral and spatial sensing. NPG Asia Mater.

[15] Wu Q, Cao J, Wang X, Liu Y, Zhao Y, Wang H, Liu Y, Huang H, Liao F, Shao M, Kang Z (2021). A metal-free photocatalyst for highly efficient hydrogen peroxide photoproduction in real seawater. Nat Commun.

[16] Zheng Y, Wei H, Liang P, Xu X, Li H, Zhang C, Hu C, Zhang X, Lei B, Wong WY, Liu Y, Zhuang J (2021). Near-infrared-excited multicolor afterglow in carbon dots-based room-temperature afterglow materials. Angew Chem Int Ed.

[17] Li D, Jing P, Sun L, An Y, Shan X, Lu X, Zhou D, Shen D, Zhai Y, Qu S, Zbořil R, Rogach AL (2018). Near-infrared excitation/emission and multiphoton-induced fluorescence of carbon dots. Adv Mater.

[18] Miao R, Zhang S, Liu J, Fang Y (2017). Zinc-reduced CQDs with highly improved stability, enhanced fluorescence, and refined solid-state applications. Chem Mater.

[19] Pachfule P, Shinde D, Majumder M, Xu Q (2016). Fabrication of carbon nanorods and graphene nanoribbons from a metal-organic framework. Nat Chem.

[20] Bai J, Ma Y, Yuan G, Chen X, Mei J, Zhang L, Ren L (2019). Solvent-controlled and solvent-dependent strategies for the synthesis of multicolor carbon dots for pH sensing and cell imaging. J Mater Chem C.

[21] Jiang K, Wang Y, Gao X, Cai C, Lin H (2018). Facile, quick, and gram-scale synthesis of ultralong-lifetime room-temperature-phosphorescent carbon dots by microwave irradiation. Angew Chem Int Ed.

[22] Jin L, Li JG, Liu LY, Wang ZL, Zhang XC (2018). Facile synthesis of carbon dots with superior sensing ability. Appl Nanosci.

[23] Liu H, Yang J, Li Z, Xiao L, Aryee AA, Sun Y, Yang R, Meng H, Qu L, Lin Y, Zhang X (2019). Hydrogen-bond-induced emission of carbon dots for wash-free nucleus imaging. Anal Chem.

[24] Song SY, Liu KK, Wei JY, Lou Q, Shang Y, Shan CX (2019). Deep-ultraviolet emissive carbon nanodots. Nano Lett.

[25] Li G, Liu C, Zhang X, Luo P, Lin G, Jiang W (2021). Highly photoluminescent carbon dots-based immunosensors for ultrasensitive detection of aflatoxin M1 residues in milk. Food Chem.

[26] Vallan L, Urriolabeitia EP, Ruipérez F, Matxain JM, Canton-Vitoria R, Tagmatarchis N, Benito AM, Maser WK (2018). Supramolecular-enhanced charge transfer within entangled polyamide chains as the origin of the universal blue fluorescence of polymer carbon dots. J Am Chem Soc.

[27] Wang Z, Liu Y, Zhen S, Li X, Zhang W, Sun X, Xu B, Wang X, Gao Z, Meng X (2020). Gram-scale synthesis of 41% efficient single-component white-light-emissive carbonized polymer dots with hybrid fluorescence/phosphorescence for white light-emitting diodes. Adv Sci.

[28] Ding H, Yu SB, Wei JS, Xiong HM (2016). Full-color light-emitting carbon dots with a surface-state-controlled luminescence mechanism. ACS Nano.

[29] Sun Y, Qin H, Geng X, Yang R, Qu L, Kani AN, Li Z (2020). Rational design of far-red to near-infrared emitting carbon dots for ultrafast lysosomal polarity imaging. ACS Appl Mater Interfaces.

[30] Feng Y, Wang Q, He M, Zhang X, Liu X, Zhao C (2019). Antibiofouling zwitterionic gradational membranes with moisture retention capability and sustained antimicrobial property for chronic wound infection and skin regeneration. Biomacromol.

[31] Ta Q, Ting J, Harwood S, Browning N, Simm A, Ross K, Olier I, Al-Kassas R (2021). Chitosan nanoparticles for enhancing drugs and cosmetic components penetration through the skin. Eur J Pharm Sci.

[32] Xu L, Zhang Y, Pan H, Xu N, Mei C, Zhang W, Cai J, Xu C (2020). Preparation and performance of radiata-pine-derived polyvinyl alcohol/carbon quantum dots fluorescent films. Materials.

[33] Hu J, Yang R, Qin H, Sun Y, Qu L, Li Z (2021). Spying on the polarity dynamics during wound healing of zebrafish by using rationally designed carbon dots. Adv Healthc Mater.

[34] Pierrat P, Wang R, Kereselidze D, Lux M, Didier P, Kichler A, Pons F, Lebeau L (2015). Efficient in vitro and in vivo pulmonary delivery of nucleic acid by carbon dot-based nanocarriers. Biomaterials.

[35] Singh V, Kashyap S, Yadav U, Srivastava A, Singh AV, Singh RK, Singh SK, Saxena PS (2019). Nitrogen doped carbon quantum dots demonstrate no toxicity under in vitro conditions in a cervical cell line and in vivo in Swiss albino mice. Toxicol Res.

[36] He Q, Zhang Z, Gao F, Li Y, Shi J (2011). In vivo biodistribution and urinary excretion of mesoporous silica nanoparticles: Effects of particle size and PEGylation. Small.

[37] Lei Z, Zhu W, Zhang X, Wang X, Wu P (2021). Bio-inspired ionic skin for theranostics. Adv Funct Mater.

[38] Zhu S, Wang L, Zhou N, Zhao X, Song Y, Maharjan S, Zhang J, Lu L, Wang H, Yang B (2014). The crosslink enhanced emission (CEE) in non-conjugated polymer dots: from the photoluminescence mechanism to the cellular uptake mechanism and internalization. Chem Commun.

[39] Lu S, Sui L, Liu J, Zhu S, Chen A, Jin M, Yang B (2017). Near-infrared photoluminescent polymer-carbon nanodots with two-photon fluorescence. Adv Mater.

[40] Dong C, Xu M, Huang J, Li F, Wei P, Tedesco AC, Bi H (2020). Dynamic thermosensitive solid-state photoluminescent carbonized polymer dots as temperature-responsive switches for sensor applications. ACS Appl Nano Mater.

[41] Liu B, Chu B, Wang YL, Hu LF, Hu S, Zhang XH (2021). Carbon dioxide derived carbonized polymer dots for multicolor light-emitting diodes. Green Chem.

[42] Zou W, Chen Y, Zhang X, Li J, Sun L, Gui Z, Du B, Chen S (2018). Cytocompatible chitosan based multi-network hydrogels with antimicrobial, cell anti-adhesive and mechanical properties. Carbohydr Polym.

