# Supplementary material for: Fluorescent carbon dots with excellent moisture retention capability for moisturizing lipstick
Source: J Nanobiotechnology. 2021 Sep 30;19:299. doi: 10.1186/s12951-021-01029-6 (PMC8482577; doi:10.1186/s12951-021-01029-6)
Supplement: Supplementary file 1 — Additional file 1: Fig. S1. High-resolution TEM image of Car-CDs. Fig. S2. Raman spectra of Car-CDs (λex = 532 nm). Fig. S3. Absolute fluorescence quantum yield of Car-CDs in methanol. Fig. S4. PL emission spectra of the Car-CDs and carmine cochineal under different conditions. Fig. S5. PL emission spectra with different excitation wavelengths of Car-CDs in methanol. Fig. S6. PL lifetime of Car-CDs. Fig. S7. The relationship between hygroscopicity and time at RH = 43% a and RH = 81% b for different samples. The relationship between moisture retention and time at RH = 43% c and RH = 81% d for different samples. [file 12951_2021_1029_MOESM1_ESM.docx]

Additional file 1

**Fluorescent carbon dots with excellent** **moisture retention capability for moisturizing lipstick**

Chen Dong^1†^, Mingsheng Xu^1†^, Shuna Wang^1†^, Menghui Ma^1^, Ozioma U. Akakuru^2^, Haizhen Ding^1^, Aiguo Wu^2,*^, Zhengbao Zha^3,^^*^, Xuemei Wang^1^ and Hong Bi^1,*^

*^1^ School of Chemistry and Chemical Engineering, Key Laboratory of Environment Friendly Polymer Materials of Anhui Province, Anhui Key Laboratory of Modern Biomanufacturing, Anhui University, Hefei 230601, China*

^*^Correspondence: [bihong@ahu.edu.cn](mailto:bihong@ahu.edu.cn); [aiguo@nimte.ac.cn](mailto:aiguo@nimte.ac.cn); [zbzha@hfut.edu.cn](mailto:zbzha@hfut.edu.cn).

^†^Chen Dong, Mingsheng Xu and Shuna Wang contributed equally.

**Experimental section**

**Materials and reagents**

Carmine cochineal was obtained from Zhejiang Yi Nuo Biotechnology Co., Ltd. Toner and vegetable oil was obtained from Nanjing Cao Cai Electronic Commerce Co., Ltd. *N*, *N*-Dimethylformamide, citric acid, sodium hyaluronate, bees wax and candelilla wax were purchased from Aladdin Chemicals Co. Ltd (Shanghai, China). Na_2_CO_3_, (NH_4_)_2_SO_4_, ethanol, methylene chloride, methanol and glycerol were purchased by Sinopharm Chemical Reagent Co., Ltd. (Shanghai, China). Dimethyl sulfoxide was obtained from Beijing Solarbio Science & Technology Co., Ltd. Dulbecco’s modified Eagle medium, penicillin/streptomycin, and fetal bovine serum were purchased from Hyclone. 3-(4, 5-dimethylthiazol-2-yl)-2, 5-diphenyltetrazolium bromide (MTT) was obtained from Sigma-Aldrich Chemicals. The lactate dehydrogenase (LDH) assay kit was purchased from Nanjing Jian Chen BioChem Co. HUVEC cells was obtained from ZQXZBIO, Shanghai, China. All chemicals were used as received without further purification unless otherwise specified. All water used during the experiment was ultrapure water from the microporous system.

**Synthesis of Car-CDs**

The Car-CDs were synthesized through a simple one-pot solvothermal method. Briefly, carmine cochineal (0.5 g) was first dissolved in 25 mL *N*, *N*-Dimethylformamide, and the solution was transferred into poly (tetrafluoroethylene)-lined autoclaves. After heating at 160 ^o^C in an oven for 6 h, the solution was then allowed to naturally cool to room temperature naturally. The crude products were then purified with a silica column chromatography using mixtures of methylene chloride and methanol as eluents. Further purification was conducted by dialysis using a dialysis membrane (MWCO 500) for 96 h, followed by freeze-drying to obtain a red powdered product. The obtained red powder can be dissolved in highly polar solvents, such as water, DMF and methanol, etc.

**Synthesis of Car-CDs-based moisturizing lipstick**

Toner (0.33 g) was first dissolved in vegetable oil (3.0 g), and then white bees wax (1.0 g) and candelilla wax (0.1 g) were added. After heating at 160 ^o^C for 0.5 h, then added the Car-CDs (0.1 g) powder under vigorous stirring. The mixture was then poured into a specific mold and transferred to the refrigerator (−20 ^o^C) for 10 min before the mold is removed to obtain a Car-CDs-based moisturizing lipstick.

**Moisture retention activities**

Moisture-absorption and moisture retention ability were performed using standard weighing method. Car-CDs (300 mg) was placed in a glass tube and transferred for 48 h to an oven with constant temperature and humidity (25 ^o^C, 43% or 81% relative humidity). The weights before (W_0_) and after (W_n_) the incubation were recorded by an electronic balance. Carmine cochineal, glycerol, and hyaluronic acid were used as references. The moisture-absorption rate was evaluated by the weight loss of the sample: (W_n_-W_0_)/W_0_×100. Similarly, Car-CDs aqueous solution (300 mg Car-CDs powder dissolved in 10% ultrapure water) was placed in a glass tube and transferred for 48 h to an oven with constant temperature and humidity (25 ^o^C, 43% or 81% relative humidity). The weights before (W_0_) and after (W_n_) the incubation were recorded by an electronic balance. The moisture retention rate was evaluated by the weight loss of the sample: W_n_/W_0_×100.

**Biocompatibility studies**

HUVEC cells was maintained in DMEM containing 1 % penicillin/streptomycin and 10 % FBS in a 95 % humidified atmosphere incubator (Thermo Scientific, Waltham, MA) with 5 % CO_2_ at 37 ^o^C. Typical MTT, LDH, and hemolytic assays were systemically evaluated according to standard protocols reports elsewhere by using HUVEC cells as model.

**Characterization**

The morphology of the sample was evaluated using a JEM-2100 TEM. FT-IR spectra were collected using a NEXUS-870 spectrometer. Raman spectra were collected on a laser confocal micro-Raman spectroscopy (InViaReflex, Renishaw, London, Britain). XPS was conducted with a commercial spectrophotometer (ESCALAB 250). The UV-vis spectra were obtained with a Shanghai Meipuda Spectrophotometer (UV-1800PC). PL measurements were performed on a Hitachi F-7000 fluorescence spectrophotometer. The HORIBA FLSP920 system was used to obtain the absolute quantum yield (QY) in the calibration sphere. Fluorescence lifetimes were measured using HORIBA FluoroMax-4P. Skin moisture contents were collected using skin moisture tester (CM825).

**Additional file**


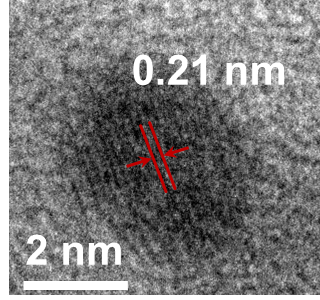


**Fig. S1.** High-resolution TEM image of Car-CDs.


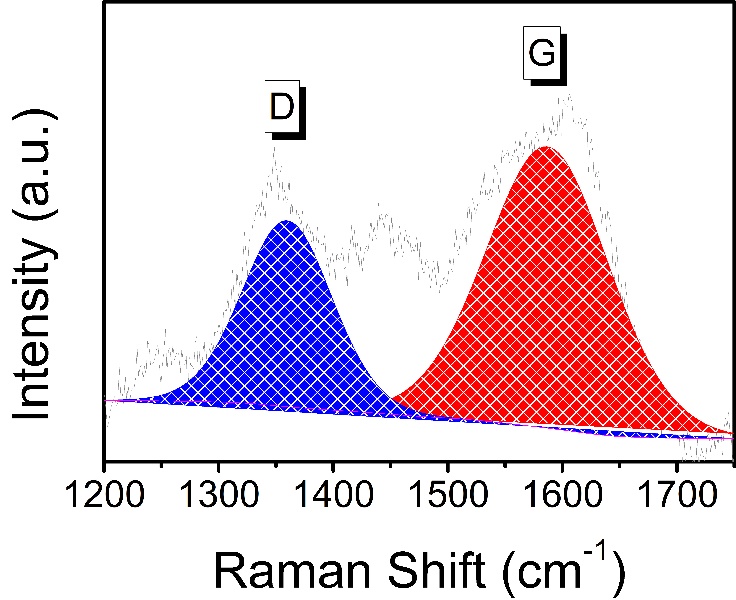


**Fig. S2.** Raman spectra of Car-CDs (*λ*_ex_ = 532 nm).


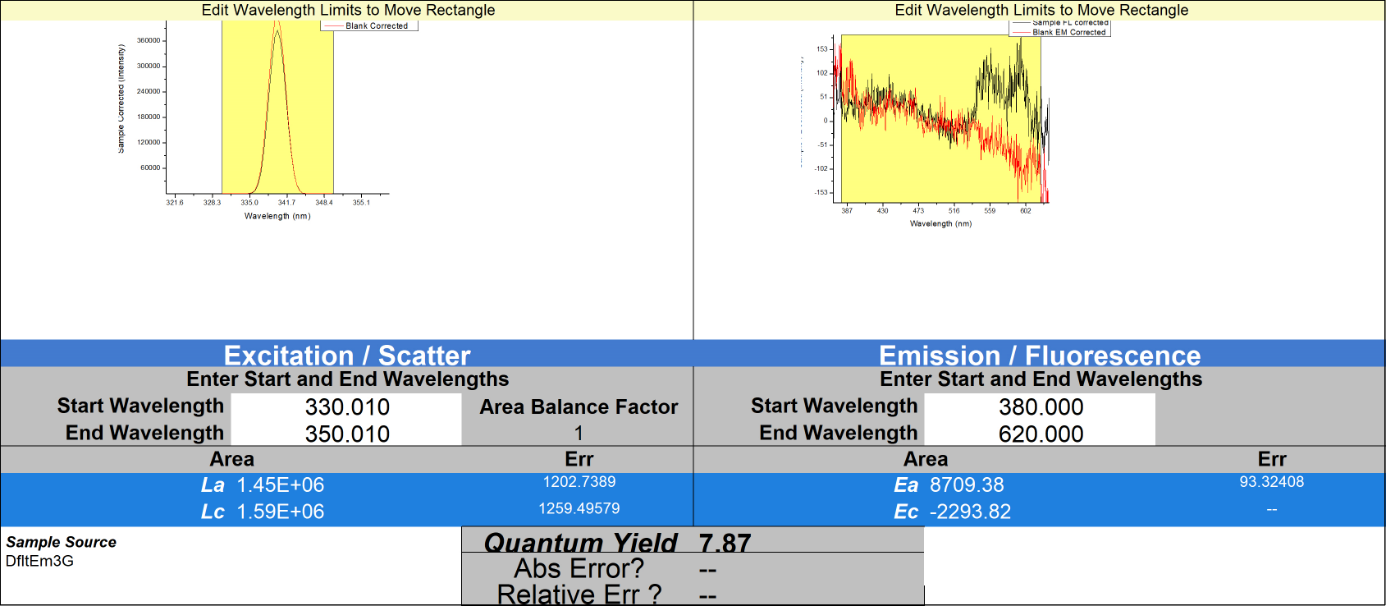


**Fig. S3.** Absolute fluorescence quantum yield of Car-CDs in methanol.


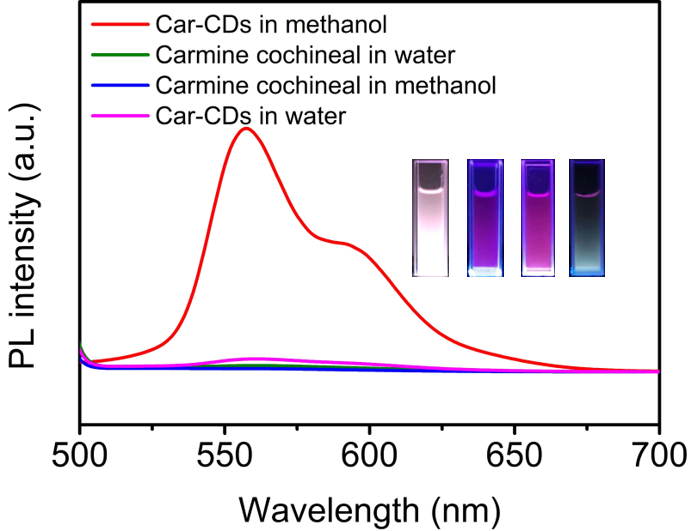


**Fig. S4.** PL emission spectra of the Car-CDs and carmine cochineal under different conditions.


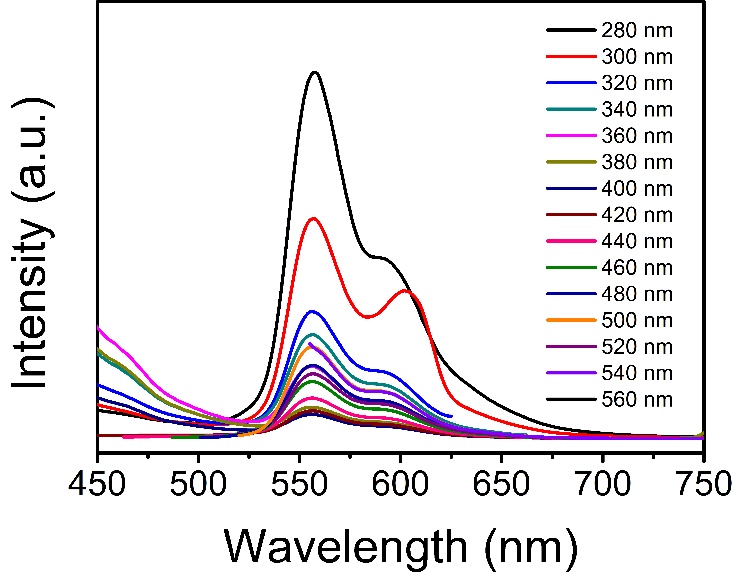


**Fig. S5.** PL emission spectra with different excitation wavelengths of Car-CDs in methanol.


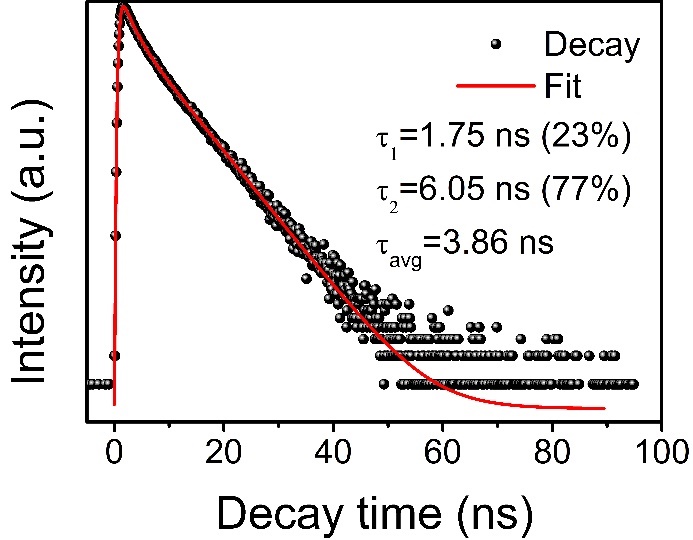


**Fig. S6.** PL lifetime of Car-CDs.


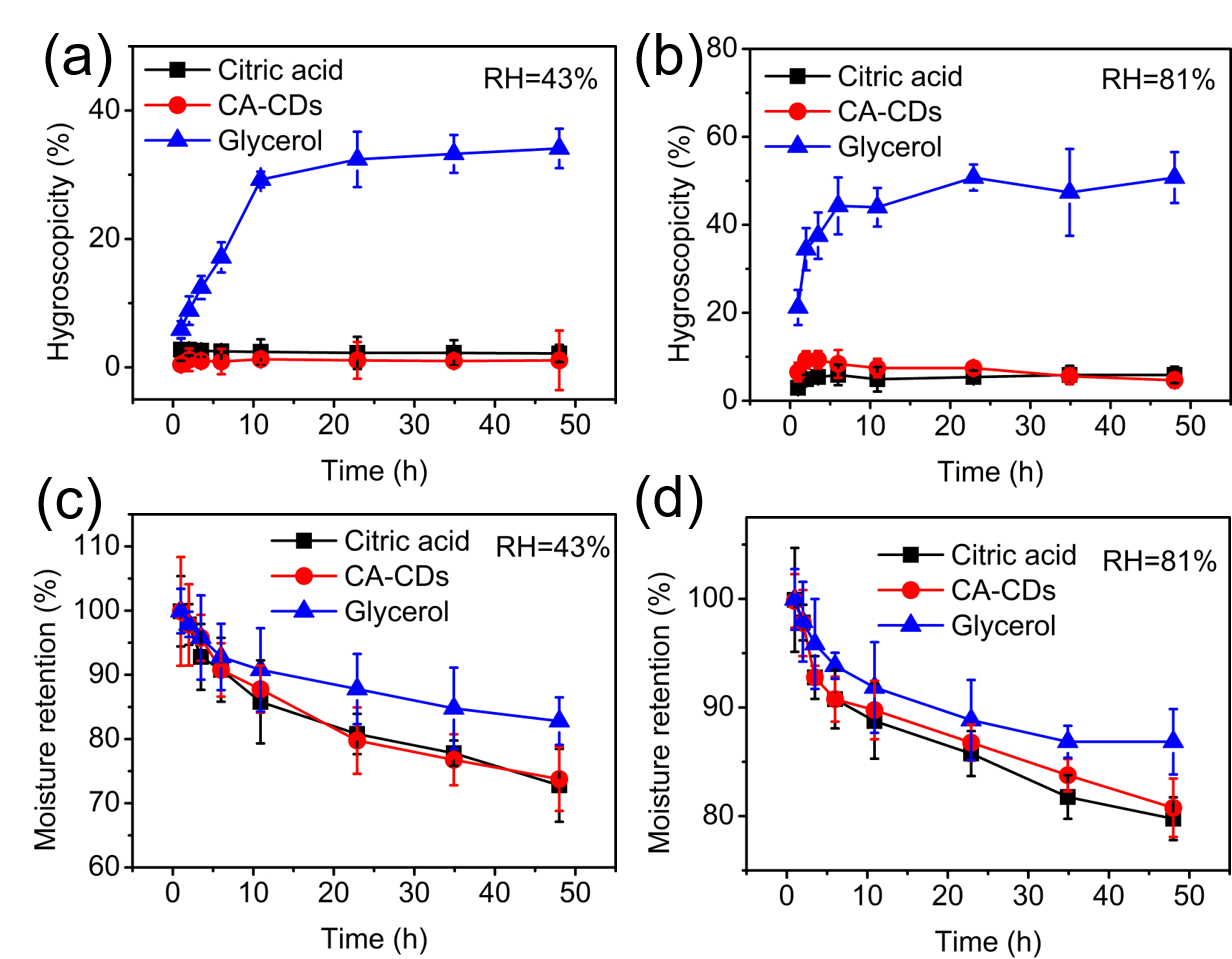


**Fig. S7.** The relationship between hygroscopicity and time at RH = 43% (a) and RH = 81% (b) for different samples. The relationship between moisture retention and time at RH = 43% (c) and RH = 81% (d) for different samples.
